# Higher dose alglucosidase alfa is associated with improved overall survival in infantile-onset Pompe disease (IOPD): data from the Pompe Registry

**DOI:** 10.1186/s13023-023-02981-2

**Published:** 2023-12-06

**Authors:** Priya S. Kishnani, David Kronn, Shugo Suwazono, Alexander Broomfield, Juan Llerena, Zuhair Nasser Al-Hassnan, Julie L. Batista, Kathryn M. Wilson, Magali Periquet, Nadia Daba, Andreas Hahn, Yin-Hsiu Chien

**Affiliations:** 1 Division of Medical Genetics, Department of Pediatrics, Duke University Medical Center, Durham, NC USA; 2 Department of Pathology and Pediatrics, New York Medical College, Valhalla, NY USA; 3 Center for Clinical Neuroscience, National Hospital Organization Okinawa National Hospital, Ginowan, Japan; 4 Willink Biochemical Genetics Unit, Manchester Center for Genomic Medicine, St Mary’s Hospital, Central Manchester Foundation Trust, Manchester, UK; 5 Centro de Genética Médica, Instituto Fernandes Figueira/FIOCRUZ, Rio de Janeiro, Brazil; 6 Department of Medical Genomics, Center for Genomic Medicine, King Faisal Specialist Hospital and Research Center, Riyadh, Saudi Arabia; 7 Sanofi, Cambridge, MA USA; 8 Navitas Data Sciences, Pottstown, PA USA; 9 Sanofi, Berlin, Germany; 10 Sanofi, Dubai, United Arab Emirates; 11 Department of Child Neurology, University Hospital Giessen, Giessen, Germany; 12 Department of Medical Genetics and Pediatrics, National Taiwan University Hospital, Taipei, Taiwan

**Keywords:** Alglucosidase alfa, Dose, Enzyme replacement therapy, Infantile onset Pompe disease, Pompe disease, Pompe registry

## Abstract

**Background:**

Studies indicate that doses of alglucosidase alfa (ALGLU) higher than label dose (20 mg/kg every other week) improve clinical outcomes in infantile-onset Pompe disease (IOPD). We investigated data from the Pompe Registry to determine the association between ALGLU dose and survival in IOPD.

**Results:**

We included 332 IOPD patients from the Registry as of January 2022 who had cardiomyopathy and were first treated at age < 1 year. We used Cox proportional hazards models to estimate hazard ratios (HR) and 95% confidence intervals (CI) for the association between ALGLU as a time-varying exposure and survival, adjusting for age at first treatment, sex, and cross-reactive immunologic material (CRIM)/immune tolerance induction (ITI) status. Dose was measured as average relative dose received over time (in multiples of label dose, range > 0 to 4 times label dose), current dose, and lagged dose. 81% patients received label dose at treatment initiation. Over time, 52% received a higher dose. Higher ALGLU dose over time was associated with improved survival: adjusted HR 0.40 (95% CI 0.22–0.73, *p* = 0.003) per 1-unit increase in average relative dose, with similar results for invasive ventilation-free survival (adjusted HR 0.48, 95% CI 0.28–0.84; *p* = 0.010). The association was consistent in patients first treated before or after 3 months of age and did not vary significantly by CRIM status. Results for current and lagged dose were similar to average dose.

**Conclusions:**

Higher ALGLU doses were associated with significantly improved overall and invasive ventilator-free survival in IOPD. Results were consistent across sensitivity analyses.

**Supplementary Information:**

The online version contains supplementary material available at 10.1186/s13023-023-02981-2.

## Background

Pompe disease (MIM# 232300) [1] is a rare, progressive, autosomal recessive lysosomal glycogen storage disorder, caused by pathogenic variants of the *GAA* gene, resulting in deficiency of lysosomal enzyme, acid α-glucosidase. Consequently, glycogen accumulation in the lysosomes results in abnormal cell functioning, causing a wide range of progressive clinical manifestations from muscle weakness to premature death [2–4]. Classic infantile-onset Pompe disease (IOPD) is characterized by severe progressive symptoms, including cardiomyopathy and muscle weakness, often causing death within first year of life. Patients rarely survive beyond 2 years without treatment [5].

With the approval of recombinant human *GAA* (rh*GAA*), also known as alglucosidase alfa (ALGLU), in 2006 for both IOPD and late-onset Pompe disease (LOPD), enzyme replacement therapy (ERT) has been the mainstay of treatment [6]. The current label dose of ALGLU is 20 mg/kg every other week (EOW) intravenously [7]. Studies have demonstrated the clinical benefits of ALGLU, including reversal of cardiomyopathy, improved motor development, ventilator-free and overall survival in IOPD [8–13]. However, studies have noted residual myopathy as well as motor and respiratory decline over time despite treatment [6, 14, 15]. In the long-term survivors of IOPD, there remains an unmet need to address the clinical plateau and potential decline in the later phases of treatment [6, 14, 15].

The quest to improve clinical outcomes, and the challenges posed by late diagnosis, has led to the investigation of whether higher doses of ALGLU offer additional benefits. Several observational studies have reported clinical benefits of higher doses of ALGLU, including benefits in gross motor function [6, 14, 16, 17], pulmonary function [6, 16, 18], and improved biomarker profile [6, 14]. However, these studies have relatively small cohorts [6, 14, 16–18]. Furthermore, in the pivotal trial, the benefits of higher dose (40 mg/kg EOW) were unclear, due to more cross-reactive immunological material (CRIM) negative patients in the higher dose group [8]. Therefore, more robust data analysis in a larger real-world patient population is warranted.

The Pompe Registry (NCT00231400) is a multinational, observational, and voluntary program that was started in 2004 to improve understanding of the natural history of the disease and clinical outcomes of real-world treatment approaches [3, 19–22]. The objective of this analysis was to use Pompe Registry data to describe the dosing patterns of ALGLU in patients with IOPD over time and to determine the potential association between ALGLU dose and clinical outcomes, including overall survival and progression to invasive ventilation.

## Methodology

### Patient population

The study population included Pompe Registry patients with a confirmed diagnosis of Pompe disease, who were determined to have IOPD, and had at least one treatment record for ALGLU, with their first treatment at < 1 year of age and having started in 2003 or later. IOPD was defined as age of symptom onset ≤ 12 months, that includes cardiac enlargement/cardiomyopathy (via echocardiogram/chest X-ray). Patients with missing date of birth or ERT initiation were excluded. Patients whose first treatment records reported a frequency “Other” (rather than weekly or EOW), or a dose > 52 mg/kg (with any frequency) were excluded. Patients whose first treatment record in the Registry was for avalglucosidase alfa, an investigational drug, or unknown drug were not included in the analysis. Registry cut-off for data analysis was January 7, 2022.

### Registry data collection

Demographic and clinical characteristics were extracted from the Registry’s electronic case report forms. These included vital status, diagnosis and treatment related information. Treatment information was recorded at initiation and then over time; this included treatment type (ALGLU, avalglucosidase alfa, investigational drug, other), dose (in mg/kg) and frequency (weekly, EOW, or other). Information regarding respiratory status, including use of non-invasive and invasive ventilation, was collected and updated over time. CRIM status was also reported. For patients with missing CRIM status, genetic data was used to impute CRIM status when possible, using *GAA* variant databases from Erasmus University [23] and Duke University [24]. Patient deaths are also reported to the Registry.

### ALGLU dose assessment

Because most patients changed doses of ALGLU over time with heterogeneous patterns of change, we analyzed dose as a time-varying exposure. We used three approaches to assess ALGLU exposure, all of which were updated over follow-up: (1) average relative dose over time, to capture treatment history, (2) current dose category based on commonly used dosing regimens, to capture current exposure, and (3) dose category 3 or 6 months in the past, referred to as a lagged analysis, to account for the possibility of dose changes made in close proximity to the outcome due to disease progression. Details of each measurement are described below.

#### Average relative dose

We calculated average dose received over time relative to label dose, as multiples of the label dose of ALGLU of 20 mg/kg EOW. A patient who always received the label dose would have an average relative dose at any time point of 1. A patient who always received a double dose, either as 20 mg/kg weekly or 40 mg/kg EOW, would have an average relative dose at any time point of 2. Patients who moved between doses would have average doses that change during follow-up, reflecting the cumulative average of the different doses up to that time point. The range for average relative dose is > 0 up to 4 (40 mg/kg weekly, quadruple the label dose, the highest dose typically used).

To calculate average relative dose, we defined one label dose-year as 1 year of ALGLU treatment at the label dose of 20 mg/kg EOW. By definition, a patient treated with the label dose for 1 year would accumulate one label dose-year of treatment, while a patient treated with double the label dose for 1 year (20 mg/kg/week or 40 mg/kg EOW) would accumulate two label dose-years in 1 year. Average relative dose at each time point was calculated as the total label dose-years received till that time divided by total years on treatment at that time, providing the average dose received over time relative to (in multiples of) the label dose. Further details regarding the conceptualization and calculation of average relative dose are presented in Additional file 1: Fig. S1.

Of note, average relative dose cannot distinguish different patterns of dosing (i.e., whether a patient received a high dose first and then the label dose or vice-versa), nor does it distinguish between dosing frequencies of weekly versus EOW, as all treatments are converted to label dose equivalents.

#### Current and lagged dose categories

For the current and lagged dose analyses, ALGLU categories were created around commonly used doses: (1) ‘none or very low dose’: interruption, discontinuation or treatment of > 0 and < 14 mg/kg/week or EOW; (2) ‘label dose’: 14 to 27 mg/kg EOW; (3) ‘40 mg/kg EOW’: > 27 to 52 mg/kg EOW; (4) ‘20 mg/kg/week’: 14 to 27 mg/kg/week; and (5) ‘40 mg/kg/week’: > 27 to 52 mg/kg/week. Categories (3) to (5) represent “higher doses” relative to the label dose.

The analysis of current dose assigned patients their current dose category, updated over time. The lagged analyses assigned patients their dose category either 3 or 6 months earlier with the aim to assess whether the results for current dose were biased due to reverse causation (e.g., patients either discontinuing treatment or moving to a higher “rescue” dose shortly before death).

### Statistical analysis

The study population was described overall, by time period of first treatment and by vital status, using descriptive statistics.

To study the association between ALGLU dose as a time-varying exposure and survival, we used Cox proportional-hazards models. Patients were followed from their first treatment until death (the primary outcome) or until their most recent follow-up record in the Registry. Patients were censored at their first report of an investigational or non-ALGLU treatment (including avalglucosidase alfa) or at the first report of a dose > 52 mg/kg weekly/biweekly, as these doses were considered implausible. Results are presented as HRs with 95% CI. A composite event of death or initiation of invasive ventilation was used as a secondary outcome. For the analysis of ventilation-free survival, patients using invasive ventilation at treatment initiation and patients without information on respiratory support were excluded.

To adjust for possible confounding by age and by age at first treatment, age (in days) was used as the time scale for all models, and age at first treatment (in weeks) was used as a stratification variable in all models. All models were also adjusted for sex, and a combined variable for CRIM status and use of ITI: CRIM-positive, CRIM-negative with ITI, CRIM-negative without ITI, and unknown CRIM status. Additional covariates were considered in sensitivity analyses as described below.

Average relative dose over time was parameterized as a continuous variable (ranging from > 0 to 4 times label dose), with the association with the outcome presented as the HR per 1-unit increase in average relative dose. We also categorized average relative dose as: ‘below label dose’: < 0.95 times label dose; ‘label dose’: 0.95 to < 1.05 times label dose; ‘between label and double dose’: 1.05 to < 1.75 times label dose; ‘double dose’: 1.75 to < 2.25 times label dose; and ‘above double to quadruple dose’: ≥ 2.25 to 4.0 times label dose. Current dose, 3-month lagged dose, and 6-month lagged dose were categorized based on commonly used doses, as described in section 5.3.2.

Sensitivity analyses included additional variables as covariates: baseline dose category (same categories as current dose), year of first treatment (2003–2010, 2010–2013, 2014–2016, 2017 or later), invasive ventilation use (yes/no, updated over time), and time from diagnosis to first treatment (≤ or > 13 days). To better reflect current treatment practices, we conducted additional sensitivity analyses restricted to patients first treated at < 6 months of age and restricted to patients first treated in 2006 or later.

We conducted analyses stratified by age at first treatment (< 3 months or ≥ 3 months) and by CRIM status (positive/negative) to examine whether the association between ALGLU dose and death was similar across these groups. We used likelihood ratio tests comparing the model with an interaction term between the stratification variable and continuous average dose to the main model without an interaction term to test the statistical significance of differences between the groups.

All statistical analyses were performed using SAS (version 9.4; SAS Institute Inc., Cary, NC). An alpha level of 0.05 was used as the criterion for statistical significance.

### Informed consent and patient privacy

Legal guardians of all patients provided informed written consent to submit their health information to the Registry, to be used for further analysis and data sharing; independent registry sites are responsible for ensuring this compliance. The Registry protocol, informed consent form, and any locally required authorization documents are reviewed and approved by the local fully constituted Institutional Review Board or Independent Ethics Committee.

## Results

### Baseline characteristics of study population

**Fig. 1:**
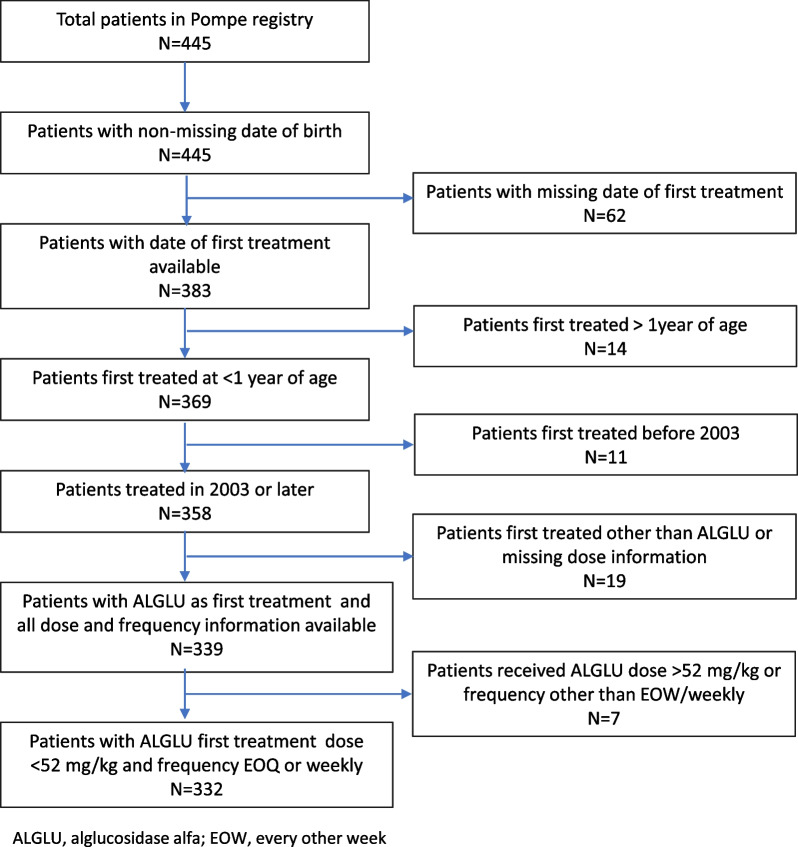
Derivation of study population from the Pompe Registry

Overall, 332 patients were included in this analysis (Fig. 1), with 1609 person-years of observation time and 88 deaths (26.5%). Patient characteristics of the full study population are described in Table 1. Median age at diagnosis of Pompe disease was 2.7 months (range 0.0–11.2). Median age at first treatment was 3.6 months (range 0.1–11.6 months). Overall, 51 patients (15.4%) were diagnosed by newborn screening (NBS). Among the 332 patients, 213 (64.2%) patients were CRIM-positive, 70 (21.1%) were CRIM-negative, and CRIM status was unknown for 49 (14.8%) patients. Immune tolerance induction (ITI) use was reported for 29 of 70 CRIM-negative patients (41.4%) and for 32 of 213 CRIM-positive patients (15.0%). At baseline, 11.3% patients used non-invasive ventilation only, 2.8% used both non-invasive and invasive ventilation, and 1.4% used invasive ventilation only. By the end of follow-up, 23.4% of patients had reported some use of non-invasive ventilation either at baseline or during follow-up, 9.6% had reported use of both non-invasive and invasive ventilation over time, and 12.8% had reported use of invasive ventilation only. Median age at death (n = 88) was 23.5 months (range 5.1–187.8). Additional characteristics of the study population are presented in Additional file 2: Table S1.

**Table 1:** Characteristics of the study population of alglucosidase alfa-treated patients with IOPD from the Pompe Registry

Characteristics	All patients
Total patients, (*n)*	332
Male, *n* (%)	160 (48.2)
Female, *n* (%)	172 (51.8)
Region (*n*)	332
EMEA, *n* (%)	88 (26.5)
JAPAC, *n* (%)	92 (27.7)
LATAM, *n* (%)	6 (1.8)
NA, *n* (%)	146 (44.0)
Age at Pompe diagnosis (months^a^) (*n*)	329
Mean (SD)	3.1 (2.77)
Median (25th, 75th percentiles)	2.7 (0.4, 5.0)
Min, max	0.0, 11.2
Diagnosed by newborn screening, *n* (%)	51 (15.4)
Age at first treatment, months (*n*)	332
Mean (SD)	3.7 (2.81)
Median (25th, 75th percentiles)	3.6 (1.1, 5.8)
Min, Max	0.1, 11.6
CRIM status (*n*)	332
Positive, *n* (%)	213 (64.2)
Negative, *n* (%)	70 (21.1)
Unknown, *n* (%)	49 (14.8)
Ever received ITI, *n (%*)	65 (19.6)
CRIM and ITI status (*n*)	332
CRIM-positive + received ITI, *n (%*)	32 (9.6)
CRIM-positive + no ITI,* n (%*)	181 (54.5)
CRIM-negative + received ITI, *n (%*)	29 (8.7)
CRIM-negative + no ITI,* n (%*)	41 (12.3)
Unknown CRIM status,* n (%*)	49 (14.8)
Patients with respiratory support data, (*n*)	282
Baseline respiratory support status (*n*)	282
None, *n (%*)	238 (84.4)
Non-invasive ventilation only, *n (%*)	32 (11.3)
Non-invasive and invasive ventilation, *n (%*)	8 (2.8)
Invasive ventilation only, *n (%*)	4 (1.4)
Ever use of respiratory support (baseline through follow-up), *n (%*)	238 (84.4)
None, *n (%*)	153 (54.3)
Non-invasive ventilation only, *n (%*)	66 (23.4)
Non-invasive and invasive ventilation, *n (%*)	27 (9.6)
Invasive ventilation only, *n (%*)	36 (12.8)
Deceased, *n* (%)	88 (26.5)
Age at death, months (*n*)	88
Mean (SD)	38.6 (39.49)
Median (25th, 75th percentiles)	23.5 (14.5, 44.5)
Min, max	5.1, 187.8
Dose related information	
Dose category at baseline^b^ (*n*)	332
Very low dose, *n (%*)	2 (0.6)
Label dose (20 mg/kg EOW), *n (%*)	270 (81.3)
40 mg/kg EOW, *n (%*)	12 (3.6)
20 mg/kg/week, *n (%*)	38 (11.4)
40 mg/kg/week, *n (%*)	10 (3.0)
Highest dose category over time^b^ (*n*)	332
Very low dose, *n (%*)	0
Label dose, *n (%*)	160 (48.2)
40 mg/kg EOW, *n (%*)	39 (11.7)
20 mg/kg/week, *n (%*)	68 (20.5)
40 mg/kg/week, *n (%*)	65 (19.6)

CRIM, cross-reactive immunological material; EMEA, Europe, the Middle East and Africa; EOW, every other week; IgG, immunoglobulin G; IOPD, infantile-onset Pompe disease; ITI, immune tolerance induction; JAPAC, Japan and Asia Pacific; LATAM, Latin America; max, maximum; min, minimum; NA, not available; SD, standard deviation

^a^Derived from the earliest of confirmatory enzyme assay date, genotype assay date, legacy diagnosis date, or date of first treatment

^b^Dose categories: Very low dose: > 0 to < 14 mg/kg EOW or weekly; Label dose: Around the label dose of 20 mg/kg EOW, range of 14 to 27 mg/kg EOW; 40 mg/kg EOW: > 27 to 52 mg/kg EOW; 20 mg/kg weekly: 14 to 27 mg/kg/week; 40 mg/kg/week: > 27 to52 mg/kg/week. Patients receiving > 52 mg/mg EOW or weekly are censored at the date of their first such dose report. 'Most recent treatment record' is the most recent record from when the patient was on treatment, prior to discontinuation for patients who discontinued treatment before the end of follow-up

The study population is described according to vital status in Additional file 2: Table S2. Deceased patients tended to be older at diagnosis and at first treatment and had longer times from first symptom to diagnosis and from diagnosis to first treatment, compared to patients alive at the end of follow-up. Deceased patients were more likely to be CRIM-negative, to use non-invasive or invasive ventilation, to have been diagnosed/treated in earlier time periods, and to have started treatment at the label dose, and were less likely to have ever received ITI or higher doses of ALGLU.

### ALGLU treatment

The initial treatment dose of ALGLU was at/about label dose for 81.3% of patients, while 18% of patients starting treatment at a higher dose: 11.4% started at/around 20 mg/kg/week, 3.6% at 40 mg/kg EOW, and 3% at 40 mg/kg/week (Table 1). Doses varied in patients over time, with about half of patients (51.8%) receiving a higher than label dose at some point during follow-up. The rest (48.2%) remained at/about label dose throughout follow-up.

Additional file 2: Table S3 describes baseline and most recent dose by categories of average relative dose. Almost all patients in the average dose category of ‘Label dose’ at the end of follow-up (n = 153) received at/around label dose at both baseline and most recent follow-up; 99.1% of the person-time in this group was in the label dose category. Patients with average dose in the category ‘Between label and double dose’ at the end of follow-up (n = 70) tended to start at label dose (88.6%), but were likely to have moved to higher doses, with only 28.5% receiving label dose or lower at their most recent record. This group spent 62.3% of total person-time in the label dose category. Patients in the average dose category of ‘Double dose’ at end of follow-up (n = 54) or ‘Above double to quadruple dose’ (n = 45) were more likely to start and end on higher than label doses. Around half of the patients in both groups were on a higher dose at baseline, and nearly all were on a higher dose at the most recent follow-up. At most recent follow-up, 80% of those in the ‘Above double to quadruple dose’ group were receiving 40 mg/kg/week, and none were at the label dose; overall, this group spent half of its person-time (49%) in the 40 mg/kg/week dose category.

### Change in clinical characteristics and dosing over time

Additional file 2: Table S4 presents detailed information on the study population by year of first treatment.

**Fig. 2:**
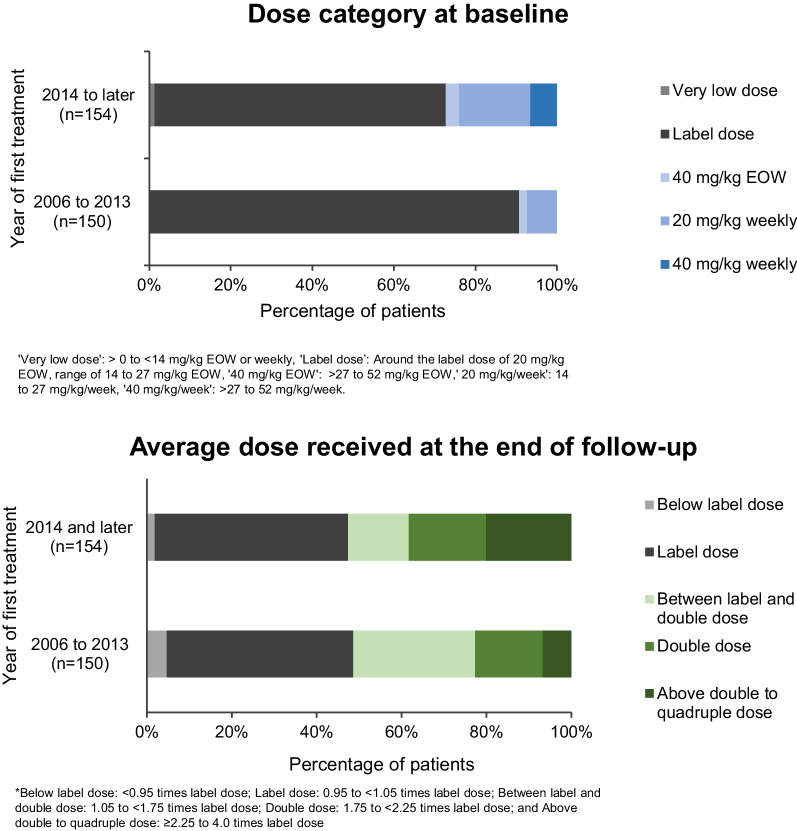
Changing trends in clinical practice: alglucosidase alfa dose over time by year of first treatment

Higher dose treatments have become more common over time (Fig. 2, Additional file 2: Table S4). Between 85 and 93% of patients began treatment at the label dose for the periods 2003–2009, and 2010–2013, compared with 67.7% during 2014–2016 and 74.2% in 2017 or later.

Additional file 2: Table S4 further describes dosing changes over time. Among higher dose regimens, 20 mg/kg/week was the most used; however, 40 mg/kg EOW and 40 mg/kg/week have become more common recently. In the 2014–2016 period, 24.6% of patients received 20 mg/kg/week initially, with 1 (1.5%) patient starting at 40 mg/kg EOW and 3 (4.6%) at 40 mg/kg/week. From 2017, the percentage starting at 20 mg/kg/week fell to 12.4%, while those receiving 40 mg/kg EOW (4.5%), and 40 mg/kg/week (7.9%) increased. Median age at first higher dose was lower for patients in the most recent periods, ranging from 24 to 43 months for patients in the first three time-periods, falling to 6.6 months for 2014–2016 and 5.4 months for 2017 and later.

Table 2 summarizes the number of events and total observation time in the study population overall and by dose at the time of event. Among 332 patients, there were 88 deaths over 1609.4 person-years (PY) of follow-up. Approximately three-quarters of patients who died were in the label dose category at the time of death, with three deaths in the 40 mg/kg EOW group and 13 deaths in the 20 mg/kg/week group. There were no deaths among patients on 40 mg/kg/week, although this dose category accounted for 9.9% of total person-time observed.

**Table 2:** Crude incidence rates of death or invasive ventilation by current and average relative dose categories

	Deaths			Composite outcome: invasive ventilation or death		
	*N* (%) events	Total PY, *N* (%)	Crude incidence rate/100 PY	*N* (%) events	Total PY, *N* (%)	Crude incidence rate/100 PY
Total events/person-time	88 (of 332 patients)	1609.4	5.47	97 (of 270 patients)	1236.8	7.84
Current dose category at the time of event^a^						
None (not currently treated)	7 (8.0)	5.7 (0.4)	121.87	4 (4.1)	4.4 (0.4)	91.71
Very low dose	0	7.9 (0.5)	0.00	1 (1.0)	7.5 (0.6)	13.30
Label dose	65 (73.9)	948.6 (58.9)	6.85	71 (73.2)	707.5 (57.2)	10.03
40 mg/kg EOW	3 (3.4)	224.6 (14.0)	1.34	7 (7.2)	184.0 (14.9)	3.80
20 mg/kg/week	13 (14.8)	262.6 (16.3)	4.95	13 (13.4)	198.7 (16.1)	6.54
40 mg/kg/week	0	160.1 (9.9)	0.00	1 (1.0)	134.7 (10.9)	0.74
Category of average relative dose over time at the time of event^b^						
Below label dose	8 (9.1)	22.8 (1.4)	35.07	5 (5.2)	21.1 (1.7)	23.71
Label dose	52 (59.1)	854.0 (53.1)	6.09	60 (61.9)	633.1 (51.2)	9.48
Between label and double dose	14 (15.9)	339.2 (21.1)	4.13	16 (16.5)	283.9 (23.0)	5.63
Double dose	12 (13.6)	249.3 (15.5)	4.81	16 (16.5)	189.1 (15.3)	8.46
Above double to quadruple dose	2 (2.3)	144.1 (9.0)	1.39	0	109.6 (8.9)	0.00

EOW, every other week; PY, person-years

^a^Dose categories: 'Very low dose': > 0 to < 14 mg/kg EOW or weekly, 'Label dose': About the label dose of 20 mg/kg EOW, range of 14 to 27 mg/kg EOW, '40 mg/kg EOW': > 27 to 52 mg/kg EOW,' 20 mg/kg/week': 14 to 27 mg/kg/week, '40 mg/kg weekly': > 27 to 52 mg/kg/week. Patients receiving > 52 mg/kg EOW or weekly are censored at the date of their first such dose report

^b^Average dose received from first treatment to the end of follow-up, in multiples of the label dose, ranging from > 0 to 4 times label dose. Average dose categories: 'Below label dose' is average dose < 0.95;'Label dose' is from 0.95 to < 1.05; 'Between label and double dose' is from 1.05 to < 1.75; 'Double dose' is 1.75 to < 2.25; 'Above double to quadruple dose' is 2.25 to 4.0

Patients receiving higher than label doses at the time of death tended to be older at death (median ages at death: 21.7 months [25th, 75th percentile: 11.7, 41.5] for the label dose group, 108.3 months [25th, 75th percentile: 20.6, 187.8] for the 40 mg/kg EOW group, and 43.5 months [25th, 75th percentile: 26.1, 63.3] for the 20 mg/kg/week group); although this is based on a small number of patients in the higher dose groups.

The overall crude (unadjusted) incidence rate of death was 5.47 per 100 PY. For patients not currently on treatment, the crude incidence rate was 121.87 per 100 PY; there were 7 deaths in this group and only 5.7 PY of observation time. This suggests that these patients discontinued treatment shortly before death. The crude incidence rate of death was lower in the three higher dose groups than in the label dose group (1.34 per 100 PY for 40 mg/kg EOW, 4.95 per 100 PY for 20 mg/kg/week, 0 for 40 mg/kg/week, vs. 6.85 per 100 PY for label dose). Patterns for the distribution of person-time and events across categories of average dose received over time were similar to those observed for current dose category at the time of event.

There were 270 patients (1236.8 PY of observation time) in the analysis of invasive ventilation-free survival, with 97 events and a crude incidence rate of invasive ventilation or death of 7.84 per 100 PY. The distribution of person-time and events across dose categories was similar to that of the main survival analysis, with approximately three-quarters of events in patients on the label dose. There were seven events (7.2%) in the 40 mg/kg EOW group and 13 (13.4%) in the 20 mg/kg/week group. There was one event in the 40 mg/kg/week category; representing 1.0% of events, while this dose category contributed 10.9% of total person-time observed. There were no composite events among patients with an average dose over time in the above double to quadruple dose category.

### Survival analysis

Table 3 presents the survival analysis for risk of death and risk of the composite event of death or invasive ventilation according to average dose received over time. Higher average dose was significantly associated with improved survival and invasive ventilation-free survival. The adjusted hazard ratio (HR) per 1-unit increase in average relative dose was 0.40 (95% confidence intervals [CI] 0.22–0.73; *p* = 0.0030). Patients with average dose above double to quadruple dose had the greatest reduction in risk of death (HR = 0.10; 95% CI 0.01–0.82, *p* = 0.03) compared to those with average dose around the label dose. Higher doses were also associated with a lower relative risk of the composite outcome, death or invasive ventilation, with an HR per 1-unit increase in average relative dose of 0.48 (95% CI 0.28–0.84; *p* = 0.010). The full model results including HR estimates for covariables are presented in Additional file 2: Table S5.

**Table 3:** Relative risk of death or invasive ventilation according to average relative dose of alglucosidase alfa

	Person Years	N Deaths	Adjusted HR^a^	95% CI	*p*-value
*Outcome: Risk of death*					
*Average relative dose over time, continuous* (range: > 0 to 4.0 times label dose)^b^					
Per 1-unit increase in average dose	1609	88	0.40	0.22, 0.73	0.0030
*Categories of average dose over time*^b^					
Below label dose	23	8	7.16	2.28, 22.48	0.0008
Label dose	854	52	1.00 (reference)	–	–
Between label and double dose	339	14	0.87	0.41, 1.82	0.7020
Double dose	249	12	0.83	0.38, 1.82	0.6340
Above double to quadruple dose	144	2	0.10	0.01, 0.82	0.0312
*Outcome: Risk of death or invasive ventilation*					
*Average relative dose over time, continuous* (range: > 0 to 4.0 times label dose)^b^					
Per 1-unit increase in average dose	1237	97	0.48	0.28, 0.84	0.0100
*Categories of average relative dose over time*^b^					
Below label dose	21	5	1.59	0.44, 5.73	0.4810
Label dose	633	60	1.00 (reference)	–	–
Between label and double dose	284	16	0.70	0.34, 1.46	0.3405
Double dose	189	16	0.96	0.47, 2.00	0.9224
Above double to quadruple dose	110	0	0.00	NE, NE	0.9895

CRIM, cross-reactive immunological material; EOW, every other week; ITI, immune tolerance induction; NE, not evaluable

^a^Models are adjusted for age (as time scale), age at first treatment, sex, and CRIM/ITI group

^b^Average dose received over time from first treatment, updated over time, measured in multiples of the label dose, ranging from > 0 to 4 times label dose. Average dose categories: 'Below label dose' is average dose < 0.95;'Label dose' is from 0.95 to < 1.05; 'Between label and double dose' is from 1.05 to < 1.75; 'Double dose' is 1.75 to < 2.25; 'Above double to quadruple dose' is 2.25 to 4.0

The association between average relative dose and risk of death was similar across sensitivity analyses (Additional file 2: Table S6). The HR for a 1-unit increase in average relative dose ranged from 0.24 to 0.47 (versus 0.40 for the main model) and remained statistically significant in models adjusted for: baseline dose category, year of first treatment, invasive ventilation use (updated over time), or time from diagnosis to first treatment, and in models restricted to patients first treated in 2006 or later or to patients treated before 6 months of age.

The model results for current dose category and 3-month and 6-month lagged dose category are presented in Additional file 2: Table S7. Few deaths in the higher dose categories resulted in wide CIs or inestimable HRs. Compared to patients on the label dose, patients receiving 40 mg/kg EOW were at significantly lower risk of death (HR 0.08, 95% CI 0.01–0.70, *p* = 0.02; n = 3 deaths). Risk was not significantly lower for those receiving 20 mg/kg/week (HR 0.85, 95% CI 0.40–1.80, *p* = 0.68; n = 13 deaths). There were no deaths in the 40 mg/kg/week group.

HRs for the 40 mg/kg EOW group were similar, and remained statistically significant, when exposure was lagged by 3 or 6 months, suggesting the current dose results were not due to reverse causation. HRs for the 20 mg/kg/week group were lower (though still not significant) after applying a 3- or 6-month lag. Thus, the current dose results for 20 mg/kg/week may be somewhat attenuated due to reverse causation, that is, patients increasing to a “rescue dose” of 20 mg/kg/week shortly before death.

Risk of death was significantly higher for patients in the none/very low dose category (HR 7.67, 95% CI 1.90–30.9, *p* = 0.004). The HR for this category was substantially lower with a 3- or 6-month lag between dose exposure and death, suggesting that the increased risk for patients currently on no/very low dose is partly due to reverse causation, that is, patients discontinuing treatment immediately prior to death.

### Sub-group analysis

Table 4 presents sub-group analyses by age at first treatment and by CRIM status. Higher average dose over time was associated with improved survival irrespective of age at first treatment. In patients first treated at < 3 months, the HR was 0.29 per 1-unit increase in average relative dose (95% CI 0.09–0.90, *p* = 0.03) and in patients first treated at ≥ 3 months, the HR was 0.43 per 1-unit increase in average relative dose (95% CI 0.21–0.87, *p* = 0.02; *p*-interaction = 0.81).

**Table 4:** Relative risk of death according to average alglucosidase alfa dose by age and CRIM status

	N patients	Person-years	N deaths	Adjusted HR^a^	95% CI	*p*-value
*Stratified by age at first treatment* (*p*-value for interaction^b^)	(0.8104)					
Age at first treatment < 3 months: Per 1-unit increase in average dose^c^	144	706	22	0.29	0.09, 0.90	0.0326
Age at first treatment ≥ 3 months: Per 1-unit increase in average dose^c^	188	726	66	0.43	0.21, 0.87	0.0195
*Stratified by CRIM status* (*p*-value for interaction^b^)	(0.7051)					
CRIM-positive: Per 1-unit increase in average dose^c^	213	1142	41	0.44	0.20, 0.98	0.0438
CRIM-negative: Per 1-unit increase in average dose^c^	70	289	29	0.24	0.04, 1.37	0.1088

CRIM, cross-reactive immunological material

^a^Models are adjusted for age (as time scale), age at first treatment, sex, and CRIM/ITI group

^b^*p*-value for interaction between the stratification variable and average dose over time, i.e., is the association between average dose and survival significantly different for the two subgroups. Calculated from likelihood ratio test comparing the main (non-stratified) model to the main model with an interaction term

^c^Average dose received over time from first treatment, updated over time, measured in multiples of the label dose. Range: > 0 to 4 times label dose

Higher average dose was associated with significantly improved survival in CRIM-positive patients (HR 0.44, 95% CI 0.20–0.98, *p* = 0.04). The HR for CRIM-negative patients suggested a lower risk of death with higher doses, though not statistically significant (HR 0.24, 95% CI 0.04–1.37, *p* = 0.11). The CRIM-negative group was relatively small, with 70 patients and 29 deaths. The *p*-value for interaction between average dose and CRIM status was not significant (*p* = 0.71), suggesting that the association of higher average dose with improved survival was not limited to one CRIM subgroup versus the other.

## Discussion

We provide results from 332 IOPD patients from the Pompe Registry to describe changes in ALGLU dosing over time and to demonstrate a robust association between higher ALGLU doses (up to four times the label dose) and improved survival and invasive ventilation-free survival. The frequency of administration of high dose ALGLU has increased over time, with more patients beginning treatment on higher doses and more patients receiving higher doses over the course of their treatment. The observed association between higher dose and improved survival was consistent across sensitivity analyses and patient subgroups and was noted irrespective of age at first treatment (before or after 3 months). All results were finely adjusted for age and age at first treatment, and were consistent across several approaches to defining dose over time. Our results are consistent with findings from several smaller studies that reported clinical benefits of higher ALGLU doses [6, 14, 16–18, 25].

Our real-world observations found that doses vary at treatment initiation and over the course of treatment, as reported in a previous real-world case series [14]. Thus, studying the association between dose and clinical outcomes required a time-varying approach that accounts for treatment history and dose changes over time. We used several approaches to assess dose over time, each with its own strengths and limitations. Average dose was used as an overall quantification of dose history. This has the advantage of considering previous treatment as well as current dose, as two patients on the same current dose may have very different total exposure to ALGLU. However, it elides differences between patients in the timing of higher versus lower doses and in treatment frequency (weekly vs. EOW). In contrast, the analysis of current dose category allows the study of treatment frequency; however, it does not account for dose history and was limited statistically by the small number of events in patients currently receiving higher doses. The 3- and 6-month lagged dose analyses allow for assessment of bias in the current dose results due to reverse causation. This could occur if patients lowered or halted their dose shortly before death, creating an artificial benefit of higher doses for survival. Alternatively, if higher doses were being used as “rescue doses” due to disease progression, the association observed between higher dose and survival would be artificially attenuated. The difference in hazard ratios between the current dose and lagged dose analyses suggested that some patients discontinued treatment shortly before death, and that other patients may have increased treatment to 20 mg/kg weekly as their disease progressed.

Data from rare disease registries can help observe changing trends in clinical scenarios, such as the impact of improved diagnostics, new treatments and evolving clinical practices. Our analysis illustrates the changing trends in clinical practice and clinical characteristics of patients with Pompe disease over time since the market availability of ALGLU (Additional file 2: Table S4). A significant reduction in the age at diagnosis, time from first symptom to diagnosis, and from diagnosis to treatment indicate improvement in diagnosis and treatment of IOPD. Diagnosis through NBS has also increased over the years, encouraging early treatment. Developments in the treatment of IOPD, such as increased use of ITI, are apparent. Similarly, the number of patients receiving higher dose ALGLU at treatment initiation has increased. Such trends indicate an inclination toward proactive treatment with higher doses of ERT at a younger age.

Our finding that higher average dose of ALGLU over time is associated with improved overall and invasive ventilation-free survival is in line with other published studies [18, 25]. A recently published study from the European Pompe Consortium in 116 patients with IOPD receiving ALGLU treatment noted a significant improvement in survival with higher dose of ALGLU versus standard label dose [25]. Similarly, in a prospective study in 18 Dutch patients with IOPD, higher dose of ALGLU (40 mg/kg/week) was associated with significant improvement in survival and ventilator-free survival, compared with standard label dose [18]. Some studies have observed potential clinical benefits of higher doses but remained uncertain, possibly due to the limited number of patients and use of higher doses only in response to clinical declines [9–11].

While in our study, the outcomes were limited to death and invasive ventilation, previous studies have demonstrated other clinical benefits of higher doses, including pharmacodynamic benefits and a decrease in biomarker levels [6, 8, 14, 16, 26, 27]. Earlier administration of higher doses has been associated with slowing gross motor decline, improved gross motor outcomes, pulmonary function, and biochemical markers [6, 8, 14, 16, 17]. Studies have also indicated improved survival (overall and invasive ventilator-free) in both CRIM-positive and CRIM-negative IOPD patients, consistent with our results [16, 26].

Our study provides the largest data analysis, to the best of our knowledge, of the association between higher ALGLU doses and treatment outcomes in IOPD. A major strength is the large heterogeneous IOPD patient population from the Pompe Registry that truly represents the real-world, in terms of variable demographic and clinical characteristics (e.g., CRIM status and ITI use) as well as treatment patterns. Furthermore, the meticulously designed statistical analysis, including three different assessments of dose and multiple sensitivity and stratified analysis, found consistent results across modelling approaches and patient subgroups. Additionally, Registry data on patients treated from the earliest availability of ALGLU to the present allowed a descriptive period-wise analysis enabling us to understand the clinical outcomes over the years as treatment patterns change.

Despite these strengths, the analysis has certain limitations. Despite a sample size of 332 patients, definitive comparisons were not possible in certain sub-groups (e.g., CRIM-negative with ITI versus without ITI, finer categories of age at first treatment, or for specific higher dose regimens) due to limited patients or events in those sub-groups. Additionally, the questions regarding ITI use were added to the Registry collection in 2019, and this information might not be updated retrospectively in some cases. This may result in under-reporting of ITI, as a lack of response was considered as ‘No’ for ITI use. The registry also does not collect reasons for dose changes in individual patients.

Examining dose patterns in real-world data is particularly challenging because patients with more severe disease tend to receive higher doses, and higher doses may be used as “rescue” doses in patients who are declining. Such bias will attenuate the observed association of higher dose with risk of death. In addition, quantifying dose over time in real-world data is challenging because each patient has their own treatment trajectory; the reasoning behind individual treatment choices is not available in population-level data, and patients cannot be cleanly grouped into a small number of treatment regimens. We attempted to address these issues using multiple analytic approaches to assess dose over time, and our results consistently show a benefit to overall and ventilation-free survival with higher ALGLU doses despite the challenges of real-world data analysis.

Finally, our analysis was limited to overall survival and invasive ventilation-free survival, and outcomes such as biomarker profiles, cardiac data and motor function status were not evaluated. In addition, while antidrug antibody (ADA) test results are available for some patients, the information was missing for many patients and hence performing a subgroup analysis for ADA was not feasible. Also, our analysis did not include examination of safety data, as this information is not collected in the Pompe Registry. Finally, though the Pompe Registry enrols patients globally, the patients included in this analysis may not be representative of all demographics.

## Conclusion

In conclusion, this data analysis from 332 patients with IOPD from the Pompe Registry showed that more patients are now receiving a higher ALGLU dose at an earlier age, and that higher ALGLU dose is associated with improved overall survival and invasive ventilator-free survival. Our study provides consistent results from a heterogenous group of patients with IOPD in the real-world setting. The findings from our study support the accumulating evidence suggesting the benefits of higher dose regimens of ALGLU in IOPD. Further data analysis focused on certain patient sub-groups may help identify those who will benefit the most from higher dose regimens of ALGLU.

### Supplementary Information

**Additional file 1: Figure S1**. Calculation of average relative dose of alglucosidase alfa over time used in survival models.

**Additional file 2: Table S1**. Additional characteristics in overall study population of alglucosidase alfa-treated patients with IOPD from the Pompe Registry. **Table S2**. Characteristics of the study population by vital status. **Table S3**. Dose categories and person-time distribution of the IOPD study population by average relative dose received at end of follow-up. **Table S4**. Patient characteristics and treatment patterns by year of first treatment. **Table S5**. Full model results for relative risk of death and of composite outcome (death or invasive ventilation) according to average alglucosidase-alfa dose over time: Adjusted hazard ratios (HR) and 95% confidence intervals (CI). **Table S6**. Sensitivity analyses for relative risk of death according to average alglucosidase alfa dose over time: Adjusted hazard ratios (HR) and 95% confidence intervals (CI). **Table S7**. Relative risk of death according to current and 3- and 6-month lagged alglucosidase alfa dose: Adjusted hazard ratios (HR) and 95% confidence intervals (CI)

## Data Availability

Data generated during this study are provided in the published article and its supplementary information files.

## Abbreviations

ADA: Antidrug antibody
ALGLU: Alglucosidase alfa
CI: Confidence intervals
CRIM: Cross-reactive immunological material
EOW: Every other week
ERT: Enzyme replacement therapy
HR: Hazard ratios
IOPD: Infantile-onset Pompe disease
ITI: Immune tolerance induction
LOPD: Late-onset Pompe disease
NBS: Newborn screening
PY: Person years
rh*GAA*: Recombinant human *GAA*
