# Supplementary material for: Higher dose alglucosidase alfa is associated with improved overall survival in infantile-onset Pompe disease (IOPD): data from the Pompe Registry
Source: Orphanet J Rare Dis. 2023 Dec 6;18:381. doi: 10.1186/s13023-023-02981-2 (PMC10698973; doi:10.1186/s13023-023-02981-2)
Supplement: Supplementary file 1 — Additional file 1: Figure S1. Calculation of average relative dose of alglucosidase alfa over time used in survival models. [file 13023_2023_2981_MOESM1_ESM.pdf]

$$\text{Average dose received at time } t = \frac{\text{Total label dose-years received to time } t}{\text{Years on treatment at time } t}$$

Patient #1:

	6 months – 1 year	1 year – 2 years	2 years – 3 years
Dose	20 mg/kg EOW	40 mg/kg EOW	40 mg/kg EOW
Label dose-years in each time period:	0.5 label dose-yrs	2 label dose-yrs	2 label dose-yrs

Age: 6 months, 1 year, 2 years, 3 years

Average dose at 1, 2, & 3 years:

Average dose at 1 yr: $\frac{\text{0.5 label dose-yrs}}{\text{0.5 yrs on treatment}} = \mathbf{1}$

Average dose at 2 yrs: $\frac{\text{2.5 label dose-yrs}}{\text{1.5 yrs on treatment}} = \mathbf{1.67}$

Average dose at 3 yrs: $\frac{\text{4.5 label dose-yrs}}{\text{2.5 yrs on treatment}} = \mathbf{1.8}$

Patient #2:

	6 months – 1.5 years	1.5 years – 2 years	2 years – 3 years
Dose	40 mg/kg EOW	20 mg/kg EOW	40 mg/kg weekly
Label dose-years in each time period:	1 label dose-yr (6 months – 1 year); 1 label dose-yr (1 year – 1.5 years)	0.5 label dose-yrs	4 label dose-yrs

Age: 6 months, 1 year, 1.5 years, 2 years, 3 years

Average dose at 1, 2, & 3 years:

Average dose at 1 yr: $\frac{\text{1 label dose-yr}}{\text{0.5 yrs on treatment}} = \mathbf{2}$

Average dose at 2 yrs: $\frac{\text{2.5 label dose-yrs}}{\text{1.5 yrs on treatment}} = \mathbf{1.67}$

Average dose at 3 yrs: $\frac{\text{6.5 label dose-yrs}}{\text{2.5 yrs on treatment}} = \mathbf{2.6}$

Note

1. The calculation at 2 years for patient #1 would be the same if the patient were on 20 mg/kg weekly from 1 to 2 years instead of 40 mg/kg EOW – either frequency would be 2 label dose-years of treatment.
2. Average dose at 2 years is the same for patient #1 and patient #2, though the timing of their higher dose periods is different. The difference in their average doses at 1 year reflects this difference in timing of doses.
