# Supplementary material for: Higher dose alglucosidase alfa is associated with improved overall survival in infantile-onset Pompe disease (IOPD): data from the Pompe Registry
Source: Orphanet J Rare Dis. 2023 Dec 6;18:381. doi: 10.1186/s13023-023-02981-2 (PMC10698973; doi:10.1186/s13023-023-02981-2)
Supplement: Supplementary file 2 — Additional file 2: Table S1. Additional characteristics in overall study population of alglucosidase alfa-treated patients with IOPD from the Pompe Registry. Table S2. Characteristics of the study population by vital status. Table S3. Dose categories and person-time distribution of the IOPD study population by average relative dose received at end of follow-up. Table S4. Patient characteristics and treatment patterns by year of first treatment. Table S5. Full model results for relative risk of death and of composite outcome (death or invasive ventilation) according to average alglucosidase-alfa dose over time: Adjusted hazard ratios (HR) and 95% confidence intervals (CI). Table S6. Sensitivity analyses for relative risk of death according to average alglucosidase alfa dose over time: Adjusted hazard ratios (HR) and 95% confidence intervals (CI). Table S7. Relative risk of death according to current and 3- and 6-month lagged alglucosidase alfa dose: Adjusted hazard ratios (HR) and 95% confidence intervals (CI) [file 13023_2023_2981_MOESM2_ESM.docx]

**Supplemental Table 1. Additional characteristics in overall study population of alglucosidase alfa-treated patients with IOPD from the Pompe Registry**

| Characteristics | All Patients |
| --- | --- |
| Time period of first treatment (*n*) | 332 |
| 2009 or earlier, *n* (%) | 107 (32.2) |
| 2010–2013, *n* (%) | 71 (21.4) |
| 2014–2016, *n* (%) | 65 (19.6) |
| 2017 or later, *n* (%) | 89 (26.8) |
| Age at symptom onset, months^a^ (*n*) | 332 |
| Mean (SD) | 1.7 (2.19) |
| Median (25^th^, 75^th^ percentiles) | 0.7 (0.0, 3.0) |
| Min, max | 0.0, 11.0 |
| Time from first symptom to diagnosis, (months^b^) (*n*) | 329 |
| Mean (SD) | 1.5 (2.32) |
| Median (25^th^, 75^th^ percentiles) | 0.6 (0.2, 2.3) |
| Min, max | -8.4, 11.0 |
| Time from diagnosis to first treatment, months (*n*) | 329 |
| Mean (SD) | 0.7 (1.12) |
| Median (25^th^, 75^th^ percentiles) | 0.4 (0.2, 0.8) |
| Min, max | 0.0, 8.5 |
| Time from first treatment to last follow-up, months (*n*) | 332 |
| Mean (SD) | 58.2 (48.95) |
| Median (25^th^, 75^th^ percentiles) | 43.7 (17.6, 91.7) |
| Min, max | 0.1, 206.4 |
| Age at first ITI, months (*n*) | 65 |
| Mean (SD) | 6.8 (10.02) |
| Median (25^th^, 75^th^ percentiles) | 3.6 (1.0, 6.1) |
| Min, max | 0.0, 58.9 |
| Ever positive for IgG antibodies (*n*) | 332 |
| Yes, *n (%*) | 139 (41.9) |
| No, *n (%*) | 40 (12.0) |
| Unknown/Not assessed, *n (%*) | 153 (46.1) |
| Age at first non-invasive respiratory ventilation, months (*n*) | 93 |
| Mean (SD) | 19.3 (31.50) |
| Median (25^th^, 75^th^ percentiles) | 7.5 (3.0, 20.7) |
| Min, max | 0.0, 167.5 |
| Age at first invasive respiratory ventilation, months (*n*) | 63 |
| Mean (SD) | 22.1 (21.47) |
| Median (25^th^, 75^th^ percentiles) | 18.2 (5.5, 27.9) |
| Min, max | 0.0, 97.3 |
| **Dose related information** | |
| Time on baseline dose, months (*n*) | 332 |
| Mean (SD) | 14.7 (21.01) |
| Median (25^th^, 75^th^ percentiles) | 6.2 (3.2, 17.7) |
| Min, max | 0.1, 172.4 |
| Age at first higher dose, months (*n*) | 172 |
| Mean (SD) | 28.6 (33.88) |
| Median (25^th^, 75^th^ percentiles) | 11.2 (4.4, 46.4) |
| Min, max | 0.2, 181.6 |
| Average time on each dose, months (*n*) | 332 |
| Mean (SD) | 21.6 (20.34) |
| Median (25^th^, 75^th^ percentiles) | 15.4 (7.2, 30.9) |
| Min, max | 0.1, 172.4 |
| Category of average relative dose received at the end of follow-up^c^ (*n*) | 332 |
| Below label dose, *n (%*) | 10 (3.0) |
| Label dose, *n (%*) | 153 (46.1) |
| Between label and double dose, *n (%*) | 70 (21.1) |
| Double dose, *n (%*) | 54 (16.3) |
| Above double to quadruple dose, *n (%*) | 45 (13.6) |
| Number of patients who discontinued treatment, *n* (%) | 11 (3.3) |
| ^a^Derived from the earliest of (1) diagnosis date reported for ophthalmic, respiratory, gastrointestinal/hepatic, renal, or musculoskeletal symptoms, (2) date of respiratory support (first use or ongoing/change), ambulatory status (ambulatory with difficulty, ambulation lost, wheelchair use), or cardiac enlargement/myopathy diagnosed via an echocardiogram or a chest X-ray, or (3) reported age of onset of symptoms. Age at symptom onset of some patients is missing.  ^b^Negative time from the first symptom to diagnosis means derived first symptom date is after the date of diagnosis.  ^c^Average dose received from first treatment to the end of follow-up, in multiples of the label dose, ranging from >0 to 4 times label dose. Average dose categories: 'Below label dose' is average dose <0.95;'Label dose' is from 0.95 to <1.05; 'Between label and double dose' is from 1.05 to <1.75; 'Double dose' is 1.75 to <2.25; 'Above double to quadruple dose' is 2.25 to 4.0. | |
| IgG, immunoglobulin G; IOPD, infantile-onset Pompe disease; ITI, immune tolerance induction; max, maximum; min, minimum; NA, not available; SD, standard deviation | |

**Supplemental Table 2. Characteristics of the study population of alglucosidase alfa-treated patients with IOPD by vital status**

| **Characteristics** | **Alive** | **Deceased** |
| --- | --- | --- |
| Total patients (*n*) | 244 | 88 |
| Male, *n* (%) | 117 (48.0) | 43 (48.9) |
| Female, *n* (%) | 127 (52.0) | 45 (51.1) |
| Region, *n* | 244 | 88 |
| EMEA, *n* (%) | 57 (23.4) | 31 (35.2) |
| JAPAC, *n* (%) | 73 (29.9) | 19 (21.6) |
| LATAM, *n* (%) | 2 (0.8) | 4 (4.5) |
| NA, *n* (%) | 112 (45.9) | 34 (38.6) |
| Time period of first treatment (*n*) | 244 | 88 |
| 2009 or earlier | 58 (23.8) | 49 (55.7) |
| 2010–2013 | 53 (21.7) | 18 (20.5) |
| 2014–2016 | 60 (24.6) | 5 (5.7) |
| 2017 or later | 73 (29.9) | 16 (18.2) |
| Age at symptom onset, months^a^ (*n*) | 244 | 88 |
| Mean (SD) | 1.6 (2.26) | 1.8 (2.00) |
| Median (25^th^, 75^th^ percentiles) | 0.4 (0.0, 3.0) | 1.0 (0.0, 3.0) |
| Min, max | 0.0, 11.0 | 0.0, 9.0 |
| Age at Pompe diagnosis, months^b^ (*n*) | 242 | 87 |
| Mean (SD) | 2.9 (2.86) | 3.7 (2.38) |
| Median (25^th^, 75^th^ percentiles) | 2.0 (0.4, 4.9) | 4.0 (2.1, 5.5) |
| Min, max | 0.0, 11.2 | 0.0, 8.9 |
| Time from first symptom to diagnosis, months^c^ (*n*) | 242 | 87 |
| Mean (SD) | 1.3 (2.20) | 2.2 (2.50) |
| Median (25^th^, 75^th^ percentiles) | 0.5 (0.2, 1.7) | 1.1 (0.3, 3.5) |
| Min, max | -8.4, 11.0 | -3.1, 9.0 |
| Diagnosed by newborn screening (*n* [%]) | 50 (20.5) | 1 (1.1) |
| Age at first treatment, months (*n*) | 244 | 88 |
| Mean (SD) | 3.5 (2.89) | 4.6 (2.41) |
| Median (25^th^, 75^th^ percentiles) | 3.0 (0.9, 5.4) | 4.6 (2.8, 6.2) |
| Min, max | 0.1, 11.6 | 0.4, 9.9 |
| Time from diagnosis to first treatment, months (*n*) | 242 | 87 |
| Mean (SD) | 0.7 (1.21) | 0.8 (0.84) |
| Median (25^th^, 75^th^ percentiles) | 0.4 (0.1, 0.7) | 0.6 (0.2, 1.2) |
| Min, max | 0.0, 8.5 | 0.0, 3.8 |
| Time from first treatment to last follow-up, months | 244 | 88 |
| Mean (SD) | 67.5 (49.29) | 32.4 (37.64) |
| Median (25^th^, 75^th^ percentiles) | 54.6 (24.0, 102.0) | 17.6 (9.2, 39.6) |
| Min, max | 0.1, 206.4 | 0.1, 180.9 |
| Age at last follow-up, months (*n*) | 244 | 88 |
| Mean (SD) | 70.9 (49.42) | 37.0 (37.28) |
| Median (25^th^, 75^th^ percentiles) | 56.8 (31.0, 105.8) | 23.5 (14.5, 42.9) |
| Min, max | 1.7, 213.6 | 5.1, 187.8 |
| CRIM status (*n*) | 244 | 88 |
| Positive, *n* (%) | 172 (70.5) | 41 (46.6) |
| Negative, *n* (%) | 41 (16.8) | 29 (33.0) |
| Unknown, *n* (%) | 31 (12.7) | 18 (20.5) |
| CRIM status among those with known status (*n* [%]) | 213 | 70 |
| Positive, *n* (%) | 172 (80.8) | 41 (58.6) |
| Negative, *n* (%) | 41 (19.2) | 29 (41.4) |
| Ever received ITI, *n* (%) | 59 (24.2) | 6 (6.8) |
| CRIM and ITI Status, *n* (%) | 244 | 88 |
| CRIM-positive + received ITI, *n* (%) | 31 (12.7) | 1 (1.1) |
| CRIM-positive + no ITI, *n* (%) | 141 (57.8) | 40 (45.5) |
| CRIM-negative + received ITI, *n* (%) | 24 (9.8) | 5 (5.7) |
| CRIM-negative + no ITI, *n* (%) | 17 (7.0) | 24 (27.3) |
| Unknown CRIM status, *n* (%) | 31 (12.7) | 18 (20.5) |
| Ever positive for IgG antibodies (*n*) | 244 | 88 |
| Yes, *n* (%) | 114 (46.7) | 25 (28.4) |
| No, *n* (%) | 36 (14.8) | 4 (4.5) |
| Unknown/Not assessed, *n* (%) | 94 (38.5) | 59 (67.0) |
| Total patients with respiratory support data (*n*) | 210 | 72 |
| Baseline respiratory support status (*n*) | 244 | 88 |
| None, *n* (%) | 181 (86.2) | 57 (79.2) |
| Non-invasive ventilation only, *n* (%) | 23 (11.0) | 9 (12.5) |
| Non-invasive and invasive ventilation, *n* (%) | 3 (1.4) | 5 (6.9) |
| Invasive ventilation only, *n* (%) | 3 (1.4) | 1 (1.4) |
| Ever use of respiratory support (baseline through follow-up) (*n*) | 210 | 72 |
| None, *n* (%) | 122 (58.1) | 31 (43.1) |
| Non-invasive ventilation only, *n* (%) | 48 (22.9) | 18 (25.0) |
| Non-invasive and invasive ventilation, *n* (%) | 17 (8.1) | 10 (13.9) |
| Invasive ventilation only, *n* (%) | 23 (11.0) | 13 (18.1) |
| Deceased (*n*) | - | 88 (100) |
| Age at death (months) (*n*) | - | 88 |
| Mean (SD) | - | 38.6 (39.49) |
| Median (25^th^, 75^th^ percentiles) | - | 23.5 (14.5, 44.5) |
| Min, max | - | 5.1, 187.8 |
| Dose category at baseline^d^ (*n* [%]) |  |  |
| Very low dose, *n* (%) | 2 (0.8) | 0 |
| Label dose, *n* (%) | 194 (79.5) | 76 (86.4) |
| 40 mg/kg EOW, *n* (%) | 9 (3.7) | 3 (3.4) |
| 20 mg/kg/week, *n* (%) | 29 (11.9) | 9 (10.2) |
| 40 mg/kg/week, *n* (%) | 10 (4.1) | 0 |
| Time on baseline dose, months (*n*) | 244 | 88 |
| Mean (SD) | 17.1 (23.52) | 8.2 (8.67) |
| Median (25^th^, 75^th^ percentiles) | 6.4 (3.5, 21.5) | 5.8 (2.3, 11.3) |
| Min, max | 0.1, 172.4 | 0.1, 45.4 |
| Age at first higher dose, months^7^ (*n*) | 146 | 26 |
| Mean (SD) | 30.7 (34.53) | 17.2 (27.78) |
| Median (25^th^, 75^th^ percentiles) | 12.6 (4.5, 52.0) | 6.8 (4.3, 9.8) |
| Min, max | 0.2, 181.6 | 0.4, 115.3 |
| Highest dose category over time^d^ (*n*) | 244 | 88 |
| Very low dose, *n* (%) | 0 | 0 |
| Label dose, *n* (%) | 98 (40.2) | 62 (70.5) |
| 40 mg/kg EOW, *n* (%) | 34 (13.9) | 5 (5.7) |
| 20 mg/kg/week, *n* (%) | 49 (20.1) | 19 (21.6) |
| 40 mg/kg/week, *n* (%) | 63 (25.8) | 2 (2.3) |
| Number of patients who discontinued treatment, *n* (%) | 4 (1.6) | 7 (8.0) |
| ^a^Derived from the earliest of (1) diagnosis date reported for ophthalmic, respiratory, gastrointestinal/hepatic, renal, or musculoskeletal symptoms, (2) date of respiratory support (first use or ongoing/change), ambulatory status (ambulatory with difficulty, ambulation lost, wheelchair use), or cardiac enlargement/myopathy diagnosed via an echocardiogram or a chest X-ray, or (3) reported age of onset of symptoms. Age at symptom onset of some patients is missing.  ^b^Derived from the earliest of confirmatory enzyme assay date, genotype assay date, legacy diagnosis date, or date of first treatment.  ^c^Negative time from the first symptom to diagnosis means derived first symptom date is after the date of diagnosis.  ^d^Dose categories: Very low dose: >0 to <14 mg/kg EOW or weekly; Label dose: Around the label dose of 20 mg/kg EOW, range of 14 to 27 mg/kg EOW; 40 mg/kg EOW: >27 to 52 mg/kg EOW; 20 mg/kg/week: 14 to 27 mg/kg/week; 40 mg/kg weekly: >27 to52 mg/kg/week. Patients receiving >52 mg/mg EOW or weekly are censored at the date of their first such dose report. 'Most recent treatment record' is the most recent record from when the patient was on treatment, prior to discontinuation for patients who discontinued treatment before the end of follow-up | | |
| CRIM, cross-reactive immunological material; EMEA, Europe, the Middle East and Africa; EOW, every other week; IgG, immunoglobulin G; IOPD, infantile-onset Pompe disease; ITI, immune tolerance induction; JAPAC, Japan and Asia Pacific; LATAM, Latin America; max, maximum; min, minimum; NA, not available; SD, standard deviation | | |

**Supplemental Table 3. Dose categories and person-time distribution by average relative dose received at end of follow-up^a^**

|  | **Below label dose^b^** | **Label dose^b^** | **Between label and double dose^b^** | **Double dose^b^** | **Above double to quadruple dose^b^** |
| --- | --- | --- | --- | --- | --- |
| Total Patients | 10 | 153 | 70 | 54 | 45 |
| Dose category^c^ at baseline | | | | | |
| Very low dose, *n* (%) | 1 (10.0) | 0 | 0 | 1 (1.9) | 0 |
| Label dose, *n* (%) | 9 (90.0) | 152 (99.3) | 62 (88.6) | 22 (40.7) | 25 (55.6) |
| 40 mg/kg EOW, *n* (%) | 0 | 0 | 1 (1.4) | 7 (13.0) | 4 (8.9) |
| 20 mg/kg/week, *n* (%) | 0 | 1 (0.7) | 6 (8.6) | 23 (42.6) | 8 (17.8) |
| 40 mg/kg/week, *n* (%) | 0 | 0 | 1 (1.4) | 1 (1.9) | 8 (17.8) |
| Dose category^c^ at most recent treatment record | | | | | |
| Very low dose, *n* (%) | 0 | 0 | 1 (1.4) | 0 | 0 |
| Label dose, *n* (%) | 10 (100) | 148 (96.7) | 19 (27.1) | 1 (1.9) | 0 |
| 40 mg/kg EOW, *n* (%) | 0 | 4 (2.6) | 26 (37.1) | 12 (22.2) | 5 (11.1) |
| 20 mg/kg/week, *n* (%) | 0 | 1 (0.7) | 22 (31.4) | 28 (51.9) | 4 (8.9) |
| 40 mg/kg/week, *n* (%) | 0 | 0 | 2 (2.9) | 13 (24.1) | 36 (80.0) |
| Percent of total person-time spent in each dose category^c^ | | | | | |
| None, % | 22.4 | 0.1 | 0.1 | 0.0 | 0.4 |
| Very low dose, % | 0.8 | 0.2 | 0.9 | 0.4 | 0.5 |
| Label dose, % | 76.8 | 99.1 | 62.3 | 18.1 | 14.3 |
| 40 mg/kg EOW, % | 0.0 | 0.4 | 18.8 | 28.6 | 17.2 |
| 20 mg/kg/week, % | 0.0 | 0.2 | 17.5 | 42.3 | 18.5 |
| 40 mg/kg/week, % | 0.0 | 0.0 | 0.5 | 10.6 | 49.1 |
| ^a^Average dose received from first treatment to the end of follow-up, in multiples of the label dose, ranging from >0 to 4 times label dose.  ^b^Average dose categories: 'Below label dose' is average dose <0.95;'Label dose' is from 0.95 to <1.05; 'Between label and double dose' is from 1.05 to <1.75; 'Double dose' is 1.75 to <2.25; 'Above double to quadruple dose' is 2.25 to 4.0.  ^c^Dose categories: 'Very low dose': > 0 to <14 mg/kg EOW or weekly, 'Label dose': About the label dose of 20 mg/kg EOW, range of 14 to 27 mg/kg EOW, '40 mg/kg EOW': >27 to 52 mg/kg EOW,' 20 mg/kg/week': 14 to 27 mg/kg/week, '40 mg/kg/week': >27 to 52 mg/kg/week. Patients receiving >52 mg/kg EOW or weekly are censored at the date of their first such dose report. 'Most recent treatment record' is the most recent record from when the patient was on treatment, prior to discontinuation for patients who discontinued treatment before the end of follow-up. | | | | | |
| EOW, every other week | | | | | |

**Supplemental Table 4.** **Patient characteristics and treatment patterns by year of first treatment**

|  | **2003 to 2005**  **N = 28** | **2006 to 2009**  **N = 79** | **2010 to 2013**  **N = 71** | **2014 to 2016**  **N = 65** | **2017 or later**  **N = 89** |
| --- | --- | --- | --- | --- | --- |
| **Overall characteristics** | | | | | |
| Age at symptom onset, months (*n*)^a^ | 28 | 79 | 71 | 65 | 89 |
| Mean (SD) | 1.4 (1.80) | 1.6 (1.92) | 1.4 (1.94) | 1.6 (2.34) | 2.1 (2.56) |
| Median (25^th^, 75^th^ percentiles) | 0.7 (0.0, 2.0) | 0.9 (0.0, 2.7) | 0.1 (0.0, 2.6) | 0.3 (0.0, 3.0) | 1.0 (0.0, 3.0) |
| Min, max | 0.0, 6.6 | 0.0, 6.8 | 0.0, 9.0 | 0.0, 11.0 | 0.0, 11.0 |
| Age at Pompe diagnosis, months (*n*)^b^ | 28 | 78 | 69 | 65 | 89 |
| Mean (SD) | 3.7 (2.62) | 3.3 (2.51) | 2.8 (2.75) | 3.0 (2.81) | 3.1 (3.01) |
| Median (25^th^, 75^th^ percentiles) | 3.9 (1.6, 5.5) | 2.9 (0.8, 5.0) | 2.3 (0.3, 4.5) | 2.6 (0.4, 5.2) | 2.3 (0.4, 5.0) |
| Min, max | 0.0, 9.0 | 0.0, 9.5 | 0.0, 11.0 | 0.0, 11.1 | 0.0, 11.2 |
| Time from first symptom to diagnosis, months (*n*)^c^ | 28 | 78 | 69 | 65 | 89 |
| Mean (SD) | 2.5 (2.12) | 1.9 (2.84) | 1.4 (2.14) | 1.3 (2.43) | 1.0 (1.74) |
| Median (25^th^, 75^th^ percentiles) | 2.5 (0.1, 4.2) | 1.0 (0.3, 3.1) | 0.5 (0.2, 2.0) | 0.7 (0.3, 1.7) | 0.4 (0.1, 1.1) |
| Min, max | -0.5, 6.0 | -8.4, 11.0 | -3.7, 8.0 | -4.8, 8.6 | -1.6, 7.5 |
| Diagnosed by newborn screening, *n* (%) | 0 | 7 (8.9) | 9 (12.7) | 11 (16.9) | 24 (27.0) |
| Age at first treatment, months (*n*) | 28 | 79 | 71 | 65 | 89 |
| Mean (SD) | 5.1 (2.44) | 3.8 (2.50) | 3.3 (2.79) | 3.7 (2.87) | 3.7 (3.07) |
| Median (25^th^, 75^th^ percentiles) | 5.3 (3.3, 6.9) | 3.9 (1.6, 5.7) | 3.0 (0.6, 5.0) | 3.3 (1.1, 6.0) | 3.2 (0.9, 5.7) |
| Min, max | 0.5, 9.3 | 0.4, 9.6 | 0.2, 11.4 | 0.1, 11.1 | 0.2, 11.6 |
| Time from diagnosis to first treatment, months (*n*) | 28 | 78 | 69 | 65 | 89 |
| Mean (SD) | 1.3 (1.09) | 0.7 (1.31) | 0.6 (0.96) | 0.8 (1.29) | 0.7 (0.87) |
| Median (25^th^, 75^th^ percentiles) | 1.0 (0.4, 2.0) | 0.4 (0.2, 0.7) | 0.3 (0.1, 0.7) | 0.4 (0.2, 1.0) | 0.4 (0.2, 0.7) |
| Min, max | 0.3, 3.9 | 0.0, 8.5 | 0.0, 6.5 | 0.0, 6.0 | 0.0, 5.0 |
| Time from first treatment to last follow-up, months | 28 | 79 | 71 | 65 | 89 |
| Mean (SD) | 89.0 (68.39) | 78.6 (59.46) | 74.2 (44.98) | 51.9 (22.72) | 22.1 (15.16) |
| Median (25^th^, 75^th^ percentiles) | 66.8 (30.6, 153.4) | 81.6 (18.8, 131.0) | 91.5 (21.0, 113.3) | 55.3 (38.4, 68.3) | 19.7 (10.2, 34.8) |
| Min, max | 3.5, 206.4 | 1.7, 184.4 | 1.3, 142.0 | 1.2, 91.8 | 0.1, 54.0 |
| Age at last follow-up, months (*n*) | 28 | 79 | 71 | 65 | 89 |
| Mean (SD) | 94.1 (68.92) | 82.4 (59.22) | 77.5 (44.36) | 55.6 (21.97) | 25.8 (15.29) |
| Median (25^th^, 75^th^ percentiles) | 74.2 (34.4, 157.9) | 82.5 (23.0, 134.7) | 93.2 (27.6, 117.3) | 56.0 (41.9, 70.4) | 21.6 (14.0, 38.0) |
| Min, max | 11.7, 213.6 | 4.5, 191.3 | 5.2, 142.9 | 8.0, 95.6 | 1.7, 54.7 |
| CRIM status (*n*) | 28 | 79 | 71 | 65 | 89 |
| Positive, *n (%*) | 18 (64.3) | 46 (58.2) | 49 (69.0) | 46 (70.8) | 54 (60.7) |
| Negative, *n (%*) | 5 (17.9) | 22 (27.8) | 16 (22.5) | 11 (16.9) | 16 (18.0) |
| Unknown, *n (%*) | 5 (17.9) | 11 (13.9) | 6 (8.5) | 8 (12.3) | 19 (21.3) |
| CRIM status among those with known status, (*n*) | 23 | 68 | 65 | 57 | 70 |
| Positive, *n (%*) | 18 (78.3) | 46 (67.6) | 49 (75.4) | 46 (80.7) | 54 (77.1) |
| Negative, *n (%*) | 5 (21.7) | 22 (32.4) | 16 (24.6) | 11 (19.3) | 16 (22.9) |
| Ever received immune modulation therapy, *n (%*) | 0 | 6 (7.6) | 7 (9.9) | 22 (33.8) | 30 (33.7) |
| CRIM and ITI Status (*n*) | 28 | 79 | 71 | 65 | 89 |
| CRIM-positive + received ITI, *n (%*) | 0 | 0 | 2 (2.8) | 15 (23.1) | 15 (16.9) |
| CRIM-positive + no ITI, *n (%*) | 18 (64.3) | 46 (58.2) | 47 (66.2) | 31 (47.7) | 39 (43.8) |
| CRIM-negative + received ITI, *n (%*) | 0 | 6 (7.6) | 4 (5.6) | 7 (10.8) | 12 (13.5) |
| CRIM-negative + no ITI, *n (%*) | 5 (17.9) | 16 (20.3) | 12 (16.9) | 4 (6.2) | 4 (4.5) |
| Unknown CRIM status, *n (%*) | 5 (17.9) | 11 (13.9) | 6 (8.5) | 8 (12.3) | 19 (21.3) |
| Ever positive for IgG antibodies (*n*) | 28 | 79 | 71 | 65 | 89 |
| Yes, *n (%*) | 12 (42.9) | 39 (49.4) | 34 (47.9) | 29 (44.6) | 25 (28.1) |
| No, *n (%*) | 0 | 8 (10.1) | 10 (14.1) | 11 (16.9) | 11 (12.4) |
| Unknown/Not assessed, *n (%*) | 16 (57.1) | 32 (40.5) | 27 (38.0) | 25 (38.5) | 53 (59.6) |
| Total patients with respiratory support data (*n*) | 27 | 77 | 67 | 60 | 51 |
| Baseline respiratory support status | 27 | 77 | 67 | 60 | 51 |
| None, *n (%*) | 25 (92.6) | 66 (85.7) | 59 (88.1) | 50 (83.3) | 38 (74.5) |
| Non-invasive ventilation only, *n (%*) | 1 (3.7) | 10 (13.0) | 4 (6.0) | 6 (10.0) | 11 (21.6) |
| Non-invasive and invasive ventilation, *n (%*) | 0 | 1 (1.3) | 3 (4.5) | 2 (3.3) | 2 (3.9) |
| Invasive ventilation, *n (%*) | 1 (3.7) | 0 | 1 (1.5) | 2 (3.3) | 0 |
| Ever use of respiratory support (baseline through follow-up) | 27 | 77 | 67 | 60 | 51 |
| None, *n (%*) | 12 (44.4) | 37 (48.1) | 37 (55.2) | 36 (60.0) | 31 (60.8) |
| Non-invasive ventilation only, *n (%*) | 5 (18.5) | 19 (24.7) | 14 (20.9) | 13 (21.7) | 15 (29.4) |
| Non-invasive and invasive ventilation, *n (%*) | 3 (11.1) | 8 (10.4) | 7 (10.4) | 5 (8.3) | 4 (7.8) |
| Invasive ventilation only, *n (%*) | 7 (25.9) | 13 (16.9) | 9 (13.4) | 6 (10.0) | 1 (2.0) |
| Deceased *n* (%) | 16 (57.1) | 33 (41.8) | 18 (25.4) | 5 (7.7) | 16 (18.0) |
| Age at death, months |  |  |  |  |  |
| Mean (SD) | 53.2 (46.84) | 45.1 (46.26) | 34.9 (33.50) | 22.1 (11.94) | 19.8 (13.08) |
| Median (25th, 75^th^ percentiles) | 41.0 (24.5, 64.0) | 23.6 (14.6, 56.4) | 21.1 (12.1, 49.5) | 20.2 (18.7, 20.5) | 16.2 (10.2, 26.7) |
| Min, max | 11.7, 187.8 | 6.3, 163.1 | 8.0, 117.0 | 9.3, 41.8 | 5.1, 52.3 |
| **Treatment Information** | | | | | |
| Dose category at baseline^d^ (*n*) | 28 | 79 | 71 | 65 | 89 |
| Very low dose, *n (%*) | 0 | 0 | 0 | 1 (1.5) | 1 (1.1) |
| Label dose, *n (%*) | 24 (85.7) | 70 (88.6) | 66 (93.0) | 44 (67.7) | 66 (74.2) |
| 40 mg/kg EOW, *n (%*) | 4 (14.3) | 2 (2.5) | 1 (1.4) | 1 (1.5) | 4 (4.5) |
| 20 mg/kg/week, *n (%*) | 0 | 7 (8.9) | 4 (5.6) | 16 (24.6) | 11 (12.4) |
| 40 mg/kg/week, *n (%*) | 0 | 0 | 0 | 3 (4.6) | 7 (7.9) |
| Age at first higher dose, months (n [%])^e^ | 13 (46.4) | 39 (49.4) | 39 (54.9) | 43 (66.2) | 38 (42.7) |
| Mean (SD) | 51.8 (52.05) | 34.9 (31.48) | 49.5 (38.52) | 16.4 (20.68) | 6.7 (6.33) |
| Median (25^th^, 75^th^ percentiles) | 38.3 (7.3, 67.1) | 24.2 (6.7, 59.5) | 42.6 (12.7, 84.1) | 6.6 (3.5, 21.3) | 5.4 (2.4, 10.1) |
| Min, max | 3.8, 181.6 | 0.7, 105.8 | 0.4, 116.9 | 0.3, 75.7 | 0.2, 35.4 |
| Highest dose category over time^d^ (*n*) | 28 | 79 | 71 | 65 | 89 |
| Very low dose, *n (%*) | 0 | 0 | 0 | 0 | 0 |
| Label dose, *n (%*) | 15 (53.6) | 40 (50.6) | 32 (45.1) | 22 (33.8) | 51 (57.3) |
| 40 mg/kg EOW, *n (%*) | 3 (10.7) | 8 (10.1) | 14 (19.7) | 5 (7.7) | 9 (10.1) |
| 20 mg/kg/week, *n (%*) | 5 (17.9) | 19 (24.1) | 17 (23.9) | 16 (24.6) | 11 (12.4) |
| 40 mg/kg/week, *n (%*) | 5 (17.9) | 12 (15.2) | 8 (11.3) | 22 (33.8) | 18 (20.2) |
| Category of average relative dose received at the end of follow-up^f^ (*n*) | 28 | 79 | 71 | 65 | 89 |
| Below label dose, *n (%*) | 0 | 4 (5.1) | 3 (4.2) | 3 (4.6) | 0 |
| Label dose, *n (%*) | 17 (60.7) | 38 (48.1) | 28 (39.4) | 19 (29.2) | 51 (57.3) |
| Between label and double dose, *n (%*) | 5 (17.9) | 19 (24.1) | 24 (33.8) | 15 (23.1) | 7 (7.9) |
| Double dose, *n (%*) | 2 (7.1) | 13 (16.5) | 11 (15.5) | 12 (18.5) | 16 (18.0) |
| Above double to quadruple dose, *n (%*) | 4 (14.3) | 5 (6.3) | 5 (7.0) | 16 (24.6) | 15 (16.9) |
| ^a^Derived from the earliest of (1) diagnosis date reported for ophthalmic, respiratory, gastrointestinal/hepatic, renal, or musculoskeletal symptoms; (2) date of respiratory support (first use or ongoing / change), ambulatory status (ambulatory with difficulty, ambulation lost, wheelchair use), or cardiac enlargement/myopathy diagnosed via an echocardiogram or a chest X-ray; or (3) reported age of onset of symptoms. Age at symptom onset of some patients is missing.  ^b^Derived from the earliest of confirmatory enzyme assay date, genotype assay date, legacy diagnosis date, or date of first treatment.  ^c^Negative time from the first symptom to diagnosis means derived first symptom date is after date of diagnosis.  ^d^Dose categories: Very low dose: > 0 to <14 mg/kg EOW or weekly; Label dose: Around the label dose of 20 mg/kg EOW, range of 14 to 27 mg/kg EOW; 40 mg/kg EOW: >27 to 52 mg/kg EOW; 20 mg/kg/week: 14 to 27 mg/kg/week; 40 mg/kg/week: >27 to 52 mg/kg/week. Patients receiving >52 mg/kg EOW or weekly are censored at the date of their first such dose report. 'Most recent treatment record' is the most recent record from when the patient was on treatment, prior to discontinuation for patients who discontinued treatment before the end of follow-up.  ^e^Earliest age in a 'higher dose' category: 40 mg/kg EOW, 20 mg/kg/week, or 40 mg/kg/week.  ^f^Average dose received from first treatment to the end of follow-up, in multiples of the label dose, ranging from >0 to 4 times label dose. Average dose categories: 'Below label dose' is average dose <0.95;'Label dose' is from 0.95 to <1.05; 'Between label and double dose' is from 1.05 to <1.75; 'Double dose' is 1.75 to <2.25; 'Above double to quadruple dose' is 2.25 to 4.0. | | | | | |
| CRIM, cross-reactive immunological material; EOW, every other week; IgG, immunoglobulin G; ITI, immune tolerance induction; max, maximum; min, minimum; NA, not available; SD, standard deviation | | | | | |

**Supplemental Table 5. Full model results:relative risk of death or invasive ventilation according to average relative dose of alglucosidase-alfa**

|  | **Person-Years** | ***N* Deaths** | **Adjusted HR^b^** | **95% CI** | **p-Value** |
| --- | --- | --- | --- | --- | --- |
| **Outcome: Risk of death** | | | | | |
| Full study population | 1609 | 88 |  |  |  |
| **Average relative dose over time, continuous^a^** (range: >0 to 4.0 times label dose) | | | | | |
| Per 1-unit increase in average dose | -- | -- | 0.40 | 0.22, 0.73 | 0.0030 |
| Sex: Male (reference) | 787 | 43 | 1.00 (ref) | -- | -- |
| Sex: Female | 823 | 45 | 0.73 | 0.44, 1.19 | 0.2051 |
| CRIM/ITI status |  |  |  |  |  |
| CRIM-positive (reference) | 1142 | 41 | 1.00 (reference) | -- | -- |
| CRIM-negative with ITI | 128 | 5 | 1.14 | 0.36, 3.64 | 0.8260 |
| CRIM-negative, no ITI | 161 | 24 | 4.40 | 2.31, 8.38 | <.0001 |
| Unknown CRIM status | 178 | 18 | 2.31 | 1.18, 4.54 | 0.0149 |
| **Categories of average relative dose over time^c^** | | | | | |
| Below label dose | 23 | 8 | 7.16 | 2.28, 22.48 | 0.0008 |
| Label dose | 854 | 52 | 1.00 (reference) | -- | -- |
| Between label and double dose | 339 | 14 | 0.87 | 0.41, 1.82 | 0.7020 |
| Double dose | 249 | 12 | 0.83 | 0.38, 1.82 | 0.6340 |
| Above double to quadruple dose | 144 | 2 | 0.10 | 0.01, 0.82 | 0.0312 |
| Sex: Male (reference) | 787 | 43 | 1.00 (reference) | -- | -- |
| Sex: Female | 823 | 45 | 0.80 | 0.48, 1.33 | 0.3905 |
| CRIM/ITI Status |  |  |  |  |  |
| CRIM-positive (reference) | 1142 | 41 | 1.00 (reference) | -- | -- |
| CRIM-negative with ITI | 128 | 5 | 1.07 | 0.33, 3.50 | 0.9106 |
| CRIM-negative, no ITI | 161 | 24 | 3.61 | 1.86, 6.99 | 0.0001 |
| Unknown CRIM status | 178 | 18 | 2.46 | 1.22, 4.97 | 0.0117 |
| **Outcome: Risk of death and invasive ventilation** | | | | | |
| Full study population | 1237 | 97 |  |  |  |
| **Average relative dose over time, continuous^a^** (range: >0 to 4.0 times label dose) | | | | | |
| Per 1-unit increase in average dose | -- | -- | 0.48 | 0.28, 0.84 | 0.0100 |
| Sex: Male (reference) | 568 | 46 | 1.00 (reference) | -- | -- |
| Sex: Female | 669 | 51 | 0.76 | 0.47, 1.22 | 0.2579 |
| CRIM/ITI status |  |  |  |  |  |
| CRIM-positive (reference) | 922 | 44 | 1.00 (reference) | -- | -- |
| CRIM-negative with ITI | 70 | 10 | 2.42 | 0.95, 6.16 | 0.0631 |
| CRIM-negative, no ITI | 128 | 28 | 3.44 | 1.85, 6.42 | <.0001 |
| Unknown CRIM status | 118 | 15 | 1.84 | 0.87, 3.88 | 0.1095 |
| **Categories of average relative dose over time^c^** | | | | | |
| Below label dose | 21 | 5 | 1.59 | 0.44, 5.73 | 0.4810 |
| Label dose | 633 | 60 | 1.00 (reference) | -- | -- |
| Between label and double dose | 284 | 16 | 0.70 | 0.34, 1.46 | 0.3405 |
| Double dose | 189 | 16 | 0.96 | 0.47, 2.00 | 0.9224 |
| Above double to quadruple dose | 110 | 0 | 0.00 | NE | 0.9895 |
| Sex: Male (reference) | 568 | 46 | 1.00 (reference) | -- | -- |
| Sex: Female | 669 | 51 | 0.78 | 0.48, 1.27 | 0.3241 |
| CRIM/ITI status |  |  |  |  |  |
| CRIM-positive (reference) | 922 | 44 | 1.00 (reference) | -- | -- |
| CRIM-negative with ITI | 70 | 10 | 2.87 | 1.02, 8.03 | 0.0448 |
| CRIM-negative, no ITI | 128 | 28 | 3.03 | 1.62, 5.65 | 0.0005 |
| Unknown CRIM status | 118 | 15 | 1.85 | 0.87, 3.94 | 0.1099 |
| ^a^Average dose received over time from first treatment, updated over time, measured in multiples of the label dose, ranging from >0 to 4 times label dose.  ^b^Models additionally adjusted for age (as time scale) and age at first treatment (as stratification variable).  ^c^Average dose categories: 'Below label dose' is average dose <0.95;'Label dose' is from 0.95 to <1.05; 'Between label and double dose' is from 1.05 to <1.75; 'Double dose' is 1.75 to <2.25; 'Above double to quadruple dose' is 2.25 to 4.0. | | | | | |
| CRIM, cross-reactive immunological material; EOW, every other week; ITI, immune tolerance induction | | | | | |

**Supplemental Table 6. Sensitivity analyses: relative risk of death according to average relative dose of alglucosidase alfa**

|  | **Person- years** | **N Deaths** | **Adjusted HR^a^** | **95% CI** | **p-Value** |
| --- | --- | --- | --- | --- | --- |
| **Average relative dose over time, continuous** (range: >0 to 4.0 times label dose) | | | | | |
| Main model results^a^  Per 1-unit increase in average dose | 1609 | 88 | 0.40 | 0.22, 0.73 | 0.0030 |
| Main model + adj. for baseline dose category^b^  Per 1-unit increase in average dose | 1609 | 88 | 0.24 | 0.09, 0.65 | 0.0052 |
| Main model + adj. for year of first treatment^c^  Per 1-unit increase in average dose | 1609 | 88 | 0.47 | 0.24, 0.93 | 0.0293 |
| Main model + adj. for invasive ventilation use (updated over time)  Per 1-unit increase in average dose | 1609 | 88 | 0.37 | 0.20, 0.69 | 0.0018 |
| Main model + adj. for time from diagnosis to 1st treatment (≤ or >13 days)  Per 1-unit increase in average dose | 1609 | 88 | 0.41 | 0.22, 0.75 | 0.0040 |
| Main model restricted to patients with 1st treatment in 2006 or later^d^  Per 1-unit increase in average dose | 1402 | 72 | 0.38 | 0.19, 0.75 | 0.0049 |
| Main model restricted to patients treated before age 6 months^e^  Per 1-unit increase in average dose | 1228 | 61 | 0.37 | 0.19, 0.74 | 0.0053 |
| ^a^All models are adjusted for sex, age at first treatment (in weeks), and CRIM/ITI status.  ^b^Baseline dose category is included in the model using the categories: <14 mg/kg weekly/EOW, 14-27 mg/kg EOW, 28-52 mg/kg EOW, 14 to 27 mg/kg/week, 28 to 52 mg/kg/week.  ^c^Year of first treatment categories: Prior to 2010, 2010–2013, 2014–2016, 2017 or later.  ^d^Population restricted to patients first treated in 2006 or later includes 304 individual patients.  ^e^Population restricted to patients treated before 6 months includes 255 individual patients. | | | | | |
| CI, confidence interval; CRIM, cross-reactive immunological material; EOW, every other week; HR, hazard ratio; ITI, immune tolerance induction | | | | | |

**Supplemental Table 7.** **Relative risk of death according to current, 3- and 6-month lagged alglucosidase alfa dose**

|  | **Person-years** | **N Deaths** | **Adjusted HR^a^** | **95% CI** | **p-value** |
| --- | --- | --- | --- | --- | --- |
| Current dose category^b^ | 1609 | 88 |  |  |  |
| None or very low dose | 14 | 7 | 7.67 | 1.90, 30.91 | 0.0042 |
| Label dose (reference) | 949 | 65 | 1.00 (reference) | -- | -- |
| 40 mg/kg EOW | 225 | 3 | 0.08 | 0.01, 0.70 | 0.0218 |
| 20 mg/kg/week | 263 | 13 | 0.85 | 0.40, 1.80 | 0.6756 |
| 40 mg/kg/week | 160 | 0 | NE | -- | -- |
|  | | | | | |
| 3-month lag between exposure and event^c^ | 1534 | 82 |  |  |  |
| Dose category |  |  |  |  |  |
| None or very low dose | 12 | 2 | 2.33 | 0.52, 10.38 | 0.2662 |
| Label dose (reference) | 907 | 62 | 1.00 (reference) | -- | -- |
| 40 mg/kg EOW | 217 | 4 | 0.14 | 0.03, 0.66 | 0.0131 |
| 20 mg/kg/week | 250 | 14 | 0.73 | 0.38, 1.39 | 0.3412 |
| 40 mg/kg/week | 148 | 0 | NE | -- | -- |
|  | | | | | |
| 6-Month lag between exposure and event^c^ | 1464 | 73 |  |  |  |
| Dose category |  |  |  |  |  |
| None or very low dose | 11 | 3 | 4.55 | 1.25, 16.60 | 0.0217 |
| Label dose (reference) | 872 | 54 | 1.00 (reference) | -- | -- |
| 40 mg/kg EOW | 209 | 3 | 0.14 | 0.03, 0.71 | 0.0172 |
| 20 mg/kg/week | 236 | 12 | 0.71 | 0.35, 1.44 | 0.3447 |
| 40 mg/kg/week | 137 | 1 | NE | -- | -- |
| ^a^All models are adjusted for sex, age at first treatment (in weeks), and CRIM/ITI status  ^b^Dose categories are as follows: 'None or very low dose': Not currently treated or > 0 to <14 mg/kg EOW or weekly, 'Label dose': Around the label dose of 20 mg/kg EOW, range of 14 to 27 mg/kg EOW, '40 mg/kg EOW': >27 to 52 mg/kg EOW,' 20 mg/kg/week': 14 to 27 mg/kg/week, '40 mg/kg/week': >27 to 52 mg/kg/week. Patients receiving >52 mg/kg EOW or weekly are censored at the date of their first such dose report.  ^c^All results are adjusted for: sex, age at first treatment (in weeks for current dose analysis, in months for lagged analysis); and CRIM/ITI status (CRIM-positive, CRIM-negative with ITI, CRIM-negative without ITI, unknown CRIM status). In the lagged analysis, risk of death is related to dosage 3 or 6 months prior to the event. | | | | | |
| CRIM, cross-reactive immunological material; EOW, every other week;ITI, immune tolerance induction | | | | | |
